# A high-intensity cluster of *Schistosoma mansoni* infection around Mbita causeway, western Kenya: a confirmatory cross-sectional survey

**DOI:** 10.1186/s41182-019-0152-y

**Published:** 2019-04-15

**Authors:** Evans Asena Chadeka, Sachiyo Nagi, Ngetich B. Cheruiyot, Felix Bahati, Toshihiko Sunahara, Sammy M. Njenga, Shinjiro Hamano

**Affiliations:** 1 Leading program, Graduate School of Biomedical Sciences, Nagasaki University, Nagasaki, Japan; 2 Department of Parasitology, Institute of Tropical Medicine (NEKKEN), Nagasaki University, Nagasaki, Japan; 3 The Joint Usage/Research Center on Tropical Diseases, Institute of Tropical Medicine (NEKKEN), Nagasaki University, Nagasaki, Japan; 4 Nagasaki University, Kenya Research Station, NUITM-KEMRI Project, Nairobi, Kenya; 5 Department of Vector Ecology and Environment, Institute of Tropical Medicine (NEKKEN), Nagasaki University, Nagasaki, Japan; 6 Eastern and Southern Africa Centre of International Parasite Control (ESACIPAC), Kenya Medical Research Institute (KEMRI), Nairobi, Kenya

**Keywords:** Schistosomiasis, Clustering, Mbita causeway, Western Kenya

## Abstract

In Kenya, communities residing along the shores and islands of Lake Victoria bear a substantial burden of schistosomiasis. Although there is a school-based deworming program in place, the transmission of *Schistosoma mansoni* varies even at a fine scale. Given the focal nature of schistosomes’ transmission, we aim to identify areas with high intensity of *S. mansoni* infection in Mbita, Homabay County, western Kenya, for prioritized integrated control measures. Our findings confirm a high intensity of *S. mansoni* infection cluster around Mbita causeway. While the current efforts to curtail morbidity due to schistosomiasis through preventive chemotherapy in schools are crucial, fine-scale mapping of risk areas is necessary for specific integrated control measures.

## Introduction

Globally, 218 million individuals suffer from schistosomiasis, while 700 million are at risk in endemic regions [1]. Among the three species commonly causing schistosomiasis, *Schistosoma mansoni*, *Schistosoma haematobium*, and *Schistosoma japonicum*, the former two are occurring in Africa [2]. Six million Kenyans are infected, and another 15 million are at risk of schistosomiasis [3]. Communities residing along the shores and on islands of Lake Victoria bear a major schistosomiasis burden in Kenya [4–8].

The Kenya national school-based deworming program (NSBDP) was launched in 2009 and schoolchildren were treated for soil-transmitted helminths (STHs). In 2012, NSBDP was scaled up nationally. Schoolchildren in endemic regions have since been treated for schistosomiasis and STHs. The national control team with cascaded support at county and sub-county levels coordinates co-administration of albendazole for STHs and praziquantel for schistosomiasis. However, some years have witnessed treatment of STHs only due to lack of praziquantel for schistosomiasis treatment [9, 10].

Considerable spatial variation in the transmission of schistosomes can be explained by human contact patterns to infested water, the presence of competent intermediate snail hosts, availability of suitable snail hosts habitats, freshwater environment contamination with stool/urine containing eggs with miracidium, and diversity of human hosts’ immunity [11]. The highly focal nature of schistosomiasis calls for fine-scale mapping of the disease. Accurate information on the distribution of the infection is necessary for prioritized allocation of constrained control resources to populations which are at a higher risk. With the devolvement of health care delivery at the county level in Kenya, neglected tropical diseases (NTD) control programs are likely to be managed at the county level. We purpose to pinpoint *S. mansoni* infection high transmission areas in Mbita Subcounty of Homabay County for prioritized intervention with a possible replication to other endemic areas.

## Methods

This study was conducted in Mbita health demographic surveillance system (HDSS), a rural setting along the shores and on islands of Lake Victoria, Homabay County, western Kenya [12]. Briefly, Mbita HDSS is comprised of Rusinga on the island and Gembe situated on the mainland. In the early 1980s, Mbita causeway was constructed to enhance linkage between Rusinga Island and mainland. The key sources of livelihoods in the area are fishing, subsistence farming, and small-scale trade.

By June 2014, Mbita HDSS data showed 5580 children (2683 boys and 2897 girls) were enrolled in preschool. We randomly sampled 1200 pre-schoolers based on sample size calculation for finite population [13]. This sample was proportionately distributed among the 66 eligible schools. In September to October 2014, we collected stool samples for two sequential days. Samples were prepared in duplicate for microscopic examination of *S. mansoni* and STHs using Kato-Katz technique [14]. *S. mansoni* infection was categorized as light (1–99 eggs per gram of feces (EPG)), moderate (100–399 EPG), or heavy (≥ 400 EPG) [15]. Pearson’s chi-square test or Fisher’s exact test was used to compare infection prevalence in the four regions of the study area. *P* value less than 0.05 was considered statistically significant. To illustrate the clustering of schistosomiasis, we utilized SaTScan software version 9 [16].

## Results

We present findings from 813 preschool children with complete parasitological data. The mean age was 5.1 years (range 2–6), 48.7% were boys while 51.3% were girls. Table 1 details the prevalence and intensity of *S. mansoni* infection and STHs. The overall prevalence of *S. mansoni* infection was 45.1% (95% CI, 41.7–48.5). There was a tendency in the decline of *S. mansoni* infection intensity with further inland location of participants’ residence from the lake, OR = 0.9989 (95% CI, 0.9987–0.9992). The prevalence of hookworms, *A. lumbricoides*, and *T. trichiura* was 1.1% (95% CI, 0.4–1.8), 1.8% (95% CI, 0.9–2.8), and 1.1% (95% CI, 0.4–1.8) respectively.

**Table 1:** Prevalence and intensity of *S. mansoni* and STHs among preschool children in Mbita, western Kenya

	Overall *n* = 813	Location				
	Prevalence (95% CI)	Gembe West (*n* = 244)	Gembe East (*n* = 186)	Rusinga West (*n* = 232)	Rusinga East (*n* = 151)	*P*
Parasite						
*S. mansoni*	45.1 (41.7–48.5)	135 (55.3%)	36 (19.4%)	124 (53.4%)	98 (64.9%)	< 0.0001^a^
Light^1^	28.9 (25.8–32.0)	60 (24.5%)	29 (15.6%)	86 (37.1%)	60 (39.7%)	< 0.0001^a^
Moderate^2^	10.6 (8.4–12.7)	28 (11.3%)	7 (3.8%)	24 (10.3%)	27 (17.9%)	< 0.0001^a^
Heavy^3^	5.7 (4.1–7.3)	21 (8.6%)	0 (0.0%)	14 (6.0%)	11 (7.3%)	< 0.0001^b^
STHs						
Hookworm	1.1 (0.4–1.8)	4 (1.6%)	2 (1.1%)	0 (0.0%)	2 (1.3%)	0.1516^b^
*A. lumbricoides*	1.8 (0.9–2.8)	5 (2.0%)	3 (1.6%)	2 (0.9%)	5 (3.3%)	0.3641^b^
*T. trichiura*	1.1 (0.4–1.8)	2 (0.8%)	2 (1.1%)	3 (1.3%)	2 (1.3%)	0.9383^b^
Residence location						
Near (< 508.1 m)^4^	406 (49.9%)	121 (49.5%)	60 (32.3%)	125 (53.9%)	103 (68.2%)	< 0.0001^a^

^1^1–99 eggs per gram of feces (EPG)

^2^100–399 EPG

^3^≥ 400 EPG

^4^Participant’s residence distance to lakeshore grouped into near, i.e., < 508.1 m based on median distance

^a^Chi-square test

^b^Fisher’s exact test

**Fig. 1:**
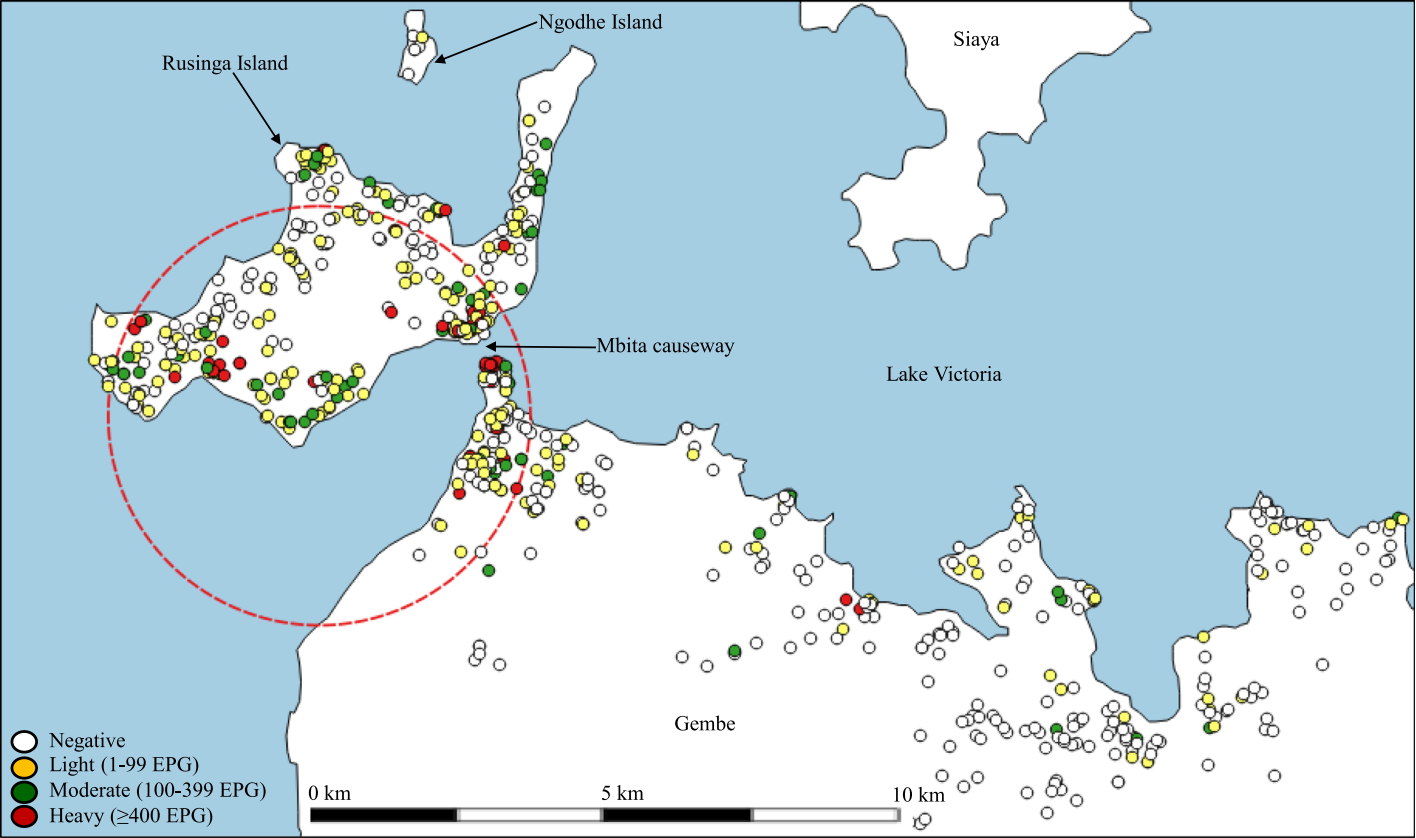
High-intensity cluster of *S. mansoni* infection among preschool children in Mbita, western Kenya. The small white, yellow, green, and red dots depict the location of negative, light, moderate, and heavy cases respectively. The red dotted circle shows the *S. mansoni* infection high-intensity cluster

Both the prevalence and intensity of *S. mansoni* infection were significantly lower in Gembe East compared to the rest of the study area. In Fig. 1, we observed a high-intensity cluster of *S. mansoni* infection covering almost the entire Rusinga Island and the western edge of Gembe close to Mbita causeway. The high-intensity cluster radius was 5.1 km containing 351 participants; the mean inside was 11.6 EPG, while mean outside was 2.5 EPG, *P* = 0.001.

## Discussion

In this report, we confirmed a high *S. mansoni* infection risk area covering Rusinga Island and the western edge of Gembe mainland around Mbita causeway (Fig. 1). We previously made this observation, and there was a trend of infection risk decreasing towards the eastern side of Gembe mainland [4]. Our previous study was however limited by the small sample obtained by cluster sampling of schools. In the current study, participants were randomly selected from all the eligible schools within the study area. Individuals residing near infested water sources are likely to be in frequent contact with infested water and hence higher probability of *S. mansoni* infection [11]. Based on the current data, the reason for high prevalence and intensity of *S. mansoni* in Gembe west and Rusinga Island is that the majority of participants were residing close to the lakeshore (55.7%) compared to those in Gembe East (32.3%). Secondly, we previously demonstrated that the high population density around Mbita causeway was associated with high *S. mansoni* infection risk. This can be explained in terms of the transmission dynamics of the parasite. In poor hygiene settings, host snails get infected with *S. mansoni* due to water environment pollution with feces containing *S. mansoni* eggs. The parasite prevalence among the host snails is dependent on absolute numbers of infected individuals and not *S. mansoni* prevalence among the human population [4]. Schistosomiasis control programs utilizing mass drug administration (MDA) with praziquantel have managed to bring down disease-related morbidity and prevalence in endemic regions. However, some settings have maintained high levels of intensity and prevalence despite MDA [17–20]. In areas where morbidity has been lowered, MDA has to be complemented with other measures including improvement of access to safe water and proper sanitation coupled with snail control to avoid resurgence of disease transmission. Fine-scale mapping of the disease transmission is paramount for identifying persistent high-risk areas and prudent allocation of limited control resources. This will be necessary as national control programs reduce morbidity, and prevalence in endemic areas and spatial patterns in turn are more heterogeneous [21].

This study had a weakness of not investigating *S. mansoni* infection in intermediate host snails and not assessing human behavioral and environmental factors associated with *S. mansoni* infection risk. Irrespective of the mentioned shortcomings, our current report re-emphasizes high schistosomiasis risk in Mbita along the shores and on islands of Lake Victoria. We have singled out the high-risk areas, and this will go a long way in prioritizing integrated control efforts based on area-specific needs geared towards reduction of *S. mansoni* morbidity and mortality.

## Abbreviations

CI: Confidence interval
EPG: Eggs per gram
HDSS: Health demographic surveillance system
MDA: Mass drug administration
NSBDP: National school-based deworming program
NTD: Neglected tropical disease
OR: Odds ratio
STH: Soil-transmitted helminth
WHO: World Health Organization
